# Clinical and epidemiologic evaluation of a 2020 chikungunya outbreak in Cambodia

**DOI:** 10.1186/s12879-022-07936-9

**Published:** 2022-12-17

**Authors:** Agus Rachmat, Gerard C. Kelly, Robert D. Hontz, Chonthida Supaprom, Vireak Heang, Phireak Hip, Jose A. Garcia-Rivera, Satharath Prom, Chhorvann Chhea, Ian W. Sutherland, Karen S. Corson, Andrew G. Letizia

**Affiliations:** 1 AC Investment Co, Contractor for NAMRU-2, Phnom Penh, Cambodia; 2 Vysnova Partners, Inc., Landover, MD USA; 3 U.S. Naval Medical Research Unit TWO, Singapore, Singapore; 4 U.S. Naval Medical Research Unit TWO, Phnom Penh, Cambodia; 5 Department of Health, Ministry of National Defense, Phnom Penh, Cambodia; 6 National Institute of Public Health, Ministry of Health, Phnom Penh, Cambodia

**Keywords:** Chikungunya, Alphavirus, Surveillance, Outbreak, Cambodia, Southeast Asia, Vector-borne disease

## Abstract

**Background:**

In 2020, the Kingdom of Cambodia experienced a nationwide outbreak of chikungunya virus (CHIKV). Despite an increase in the frequency of outbreaks and expanding geographic range of CHIKV, diagnostic challenges remain, and limited surveillance data of sufficient granularity are available to characterize epidemiological profiles and disease dynamics of the virus.

**Methods:**

An ongoing and long-standing cross-sectional study of acute undifferentiated febrile illness (AUFI) in Cambodia was leveraged to describe the disease epidemiology and characterize the clinical presentation of patients diagnosed with CHIKV during the 2020 outbreak. Participants presenting with AUFI symptoms at ten study locations provided acute and convalescent blood samples and were tested for CHIKV using a reverse transcription-polymerase chain reaction (RT-PCR) and serological diagnostic methods including IgM and IgG. Acute and follow-up clinical data were also collected.

**Results:**

From 1194 participant blood samples tested, 331 (27.7%) positive CHIKV cases were detected. Most CHIKV positive individuals (280, 84.6%) reported having a fever 3 to 4 days prior to visiting a health facility. Symptoms including chills, joint pain, nausea, vomiting, and lesions were all statistically significant among CHIKV positive participants compared to CHIKV negative AUFI participants. Cough was negatively associated with CHIKV positive participants. Positivity proportions were significantly higher among adults compared to children. No significant difference was found in positivity proportion between rainy and dry seasons during the outbreak. Positive CHIKV cases were detected in all study site provinces, with the highest test positivity proportion recorded in the rural northeast province of Kratie.

**Conclusions:**

Surveillance data captured in this study provided a clinical and epidemiological characterization of positive CHIKV patients presenting at selected health facilities in Cambodia in 2020, and highlighted the widespread distribution of the outbreak, impacting both urban and rural locations. Findings also illustrated the importance of utilizing both RT-PCR and serological testing for effective CHIKV surveillance.

**Supplementary Information:**

The online version contains supplementary material available at 10.1186/s12879-022-07936-9.

## Background

Chikungunya virus (CHIKV) is a vector-borne Alphavirus transmitted by the *Aedes* mosquito, principally *Aedes aegypti* and *Aedes albopictus* [1–3]. Endemic to tropical and sub-tropical environments, CHIKV has a large geographic distribution, with approximately 39% of the world’s population at risk of infection [3–5]. Originating in Africa and subsequently spreading across the world, CHIKV comprises three unique lineages: (1) West African, (2) Asian, and (3) East-Central-South-African, including a recently identified sub-lineage known as the Indian Ocean Lineage (ECSA-IOL) [6–8].

CHIKV generally presents as an acute febrile illness, often accompanied with skin rash, headache, nausea, and arthralgia [2, 3]. Whilst usually self-limiting and rarely fatal, CHIKV remains of global public health importance due to its potential to cause prolonged severe arthralgia, central nervous system disease, and chronic morbidity [7, 9–12], making long-term effects of this disease especially debilitating to infected individuals as measured in disability adjusted life years (DALYs). Given its widespread distribution and large populations at risk of infection, managing CHIKV transmission places significant pressure on health systems and can have lasting socio-economic consequences in impacted areas, particularly during and following outbreak events [3, 11, 12].

Since 2000, there has been an increase in frequency of large CHIKV outbreaks, including a globally significant epidemic beginning in eastern Kenya in 2004 [13] that lead to major outbreaks in countries bordering the Indian Ocean, India, and Southeast Asia [1–3]. This epidemic was largely attributed to the global spread of *Ae. albopictus* and the adaptation of the ECSA-IOL sub-lineage to this vector species. In Cambodia, clusters of the ECSA genotype were first detected in 2011, and a subsequent outbreak was detected in 2012 [14, 15]. Associated with this global epidemic was an emergence of additional clinical complications, enhanced transmission rates, and the introduction of the virus to previously non-endemic areas, including more temperate regions such as Europe [3, 16–18]. Despite this increased spread, public health surveillance systems have largely lacked capability to identify CHIKV and differentiate from other causes of an undifferentiated febrile illness, including dengue virus (DENV)—leading to the potential mis-diagnosing and under-reporting of cases [3, 19–21]. There remains a need to strengthen baseline evidence regarding universal consensus on representative clinical features that characterize CHIKV to better understand the virus and its impact on at-risk populations [3].

In 2006, a health facility-based surveillance program entitled “Surveillance and Etiology of Acute Undifferentiated Febrile Illnesses in Cambodia” [22] was established as part of a collaboration between the Royal Cambodian Ministry of Health, the Cambodian National Institute of Public Health (NIPH), and the United States (US) Naval Medical Research Unit-2 (NAMRU-2). Key objectives of this program were to implement standardized laboratory procedures for patients presenting with acute undifferentiated febrile illness (AUFI), describe the clinical and demographic characteristics of patients seeking healthcare services with AUFI, and identify the etiologies associated with AUFI among persons seeking healthcare services in Cambodia. In 2020, Cambodia experienced a resurgence of CHIKV transmission, resulting in a nationwide outbreak [23]. The objectives of this study were to evaluate surveillance data captured by the established AUFI surveillance program to describe disease epidemiology, and to characterize the clinical presentation and associated symptoms of patients diagnosed with CHIKV during the 2020 outbreak in Cambodia.

## Methods

### Study population and design

Data pertaining to the 2020 CHIKV outbreak were captured as part of a large-scale health-facility-based fever surveillance program established to capture AUFI data between December 2006 and January 2021. The study utilized a cross-sectional design in a population of patients seeking health care services for AUFI symptoms. Within this large-scale study, CHIKV data were collected at ten study site facilities in six provinces. Diagnostic and epidemiological data were collected at AUFI sites where existing laboratory specific and surveillance data collection procedures were established. These included three sites in Kandal province, two sites each in Kampong Speu and Kratie provinces, and one site each in Battambang, Preah Vihear, and Sihanoukville provinces. Study sites in Kandal and Kampong Speu provinces were classified as urban areas, with the remaining sites classified as rural.

Inclusion criteria for the study included consenting individuals aged 2 years or older presenting at a study site health facility with a measured oral or tympanic temperature ≥ 38 °C or axillary temperature ≥ 37.5 °C, a history of fever for at least 24 h but not greater than 10 days, and with an illness that was not able to be diagnosed during the physical examination or history. Individuals were excluded from the study if they did not meet the inclusion criteria or declined to participate. Patients meeting the study inclusion criteria were invited to enroll upon presentation at the participating health facilities by study coordinators and facility-based health care workers. Written informed consent was obtained from all adult patients (≥ 18 years of age) who agreed to participate, or the parent/legal guardian of non-adult participants (< 18 years of age). Written assent was obtained from non-adults aged 8–17 years of age.

Following enrollment, participants received a unique study number, completed a questionnaire, and underwent a medical history and examination by a trained healthcare worker. The questionnaire captured demographic information pertaining to the study participant including name, gender, occupation, address, and contact details. Clinical examination data were also collected using an acute clinic visit form, including onset and history of current illness, associated symptoms and duration, existing medications, travel and potential exposure history, temperature, respiratory rate, pulse rate, blood pressure, and clinical assessment. Blood samples were also collected during enrollment and sent for laboratory testing. Participants were asked to return to the health facility between 14–30 days after their initial visit for follow-up assessment. During the follow-up assessment, a targeted medical examination was conducted, a questionnaire capturing clinical information including the duration of symptoms, medication taken, and recovery status of the patient was completed, and a convalescent blood sample was collected. In the event a participant did not return for their scheduled follow-up visit, study-associated healthcare workers were sent to the participant’s home or place of work to conduct follow-up visits.

### Specimen processing, transportation, and laboratory testing

Blood samples were collected by venipuncture from patients during the acute phase, and when possible, during follow-up examinations by study laboratory technicians and sent to the NAMRU-2 laboratory in Phnom Penh. Serum was separated from whole blood then aliquoted into pre-labeled cryovials and stored at − 70 °C or liquid nitrogen.

Reverse transcription-polymerase chain reaction (RT-PCR) and immunoglobulin M (IgM)/immunoglobulin G (IgG) enzyme-linked immunosorbent assays (ELISAs) were performed on each sample. For CHIKV RT-PCR, ribonucleic acid (RNA) was extracted from serum using QIAamp viral RNA mini kits (QIAGEN, Hilden, Germany) as per the manufacturer instructions and stored at − 80 °C [24]. The detection limit of the CHIKV RT-PCR protocol was 27 synthetic RNA copies and 1.2 × 10^−2^ ID per reaction [24]. ELISA assays were analyzed and interpreted as per manufactures instructions as Positive, Equivocal, or Negative. CHIKV IgM serological assays were conducted on acute serum samples using Anti-Chikungunya virus ELISA (IgM) test kit (EI 293a-9601M, EUROIMMUN, Lubeck, Germany). CHIKV IgG serological assays were conducted on acute and convalescent serum samples using ELISA as previously described [25]. Convalescent samples found to be IgG positive were paired with the result of the respective acute sample serum to determine if a four-fold titer increase occurred. Blood samples were also tested for Dengue when possible, by RT-PCR, using similar methods [26].

### Definition of a positive chikungunya virus infection

For this study, a confirmed positive CHIKV case was defined as a participant who had a sample that tested positive for either RT-PCR, IgM acute positive, or IgG seroconversion in a paired acute-convalescent serum sample (four-fold or greater increase in titer).

### Data management and statistical analysis

All demographic, clinical, epidemiological, and laboratory data were de-identified using the participant study number and double-entered into a MS Access^®^ (Microsoft Inc., Redmond, WA, USA) relational database by trained data-entry staff. Data were analyzed using Stata software (StataCorp, College Station, TX, USA). Categorical variables were analyzed using Chi-square statistics and logistic regression models used to calculate odds ratio (OR) and 95% confidence intervals (CIs). A significance level of p < 0.05 was set for all statistical tests.

## Results

**Fig. 1:**
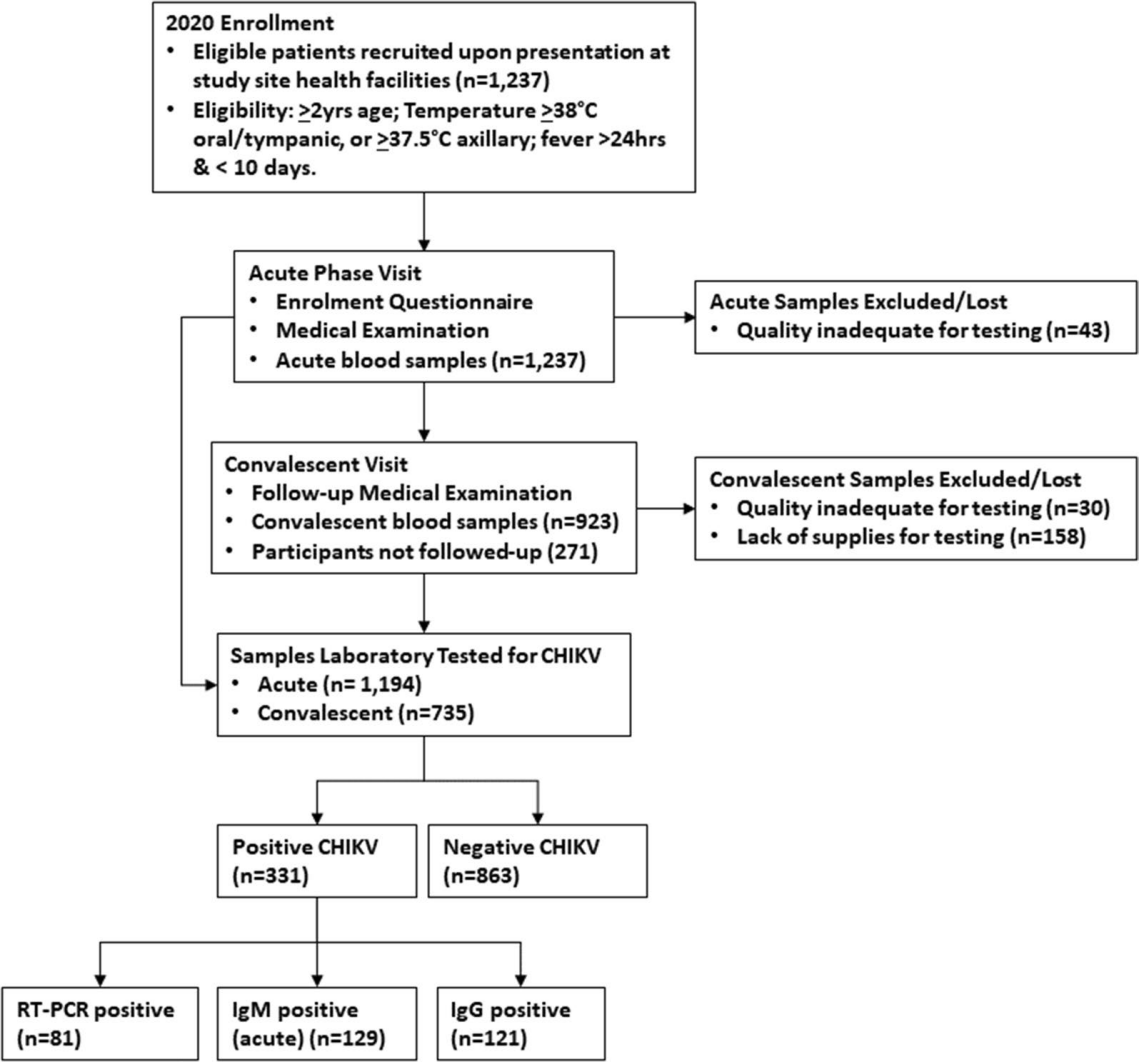
Flow chart illustrating participant enrolment, acute and convalescent visits, and associated blood samples collected and tested

A total of 1237 AUFI patients were enrolled into the AUFI study during 2020. Within this cohort, acute blood samples taken from 43 individuals (3.5%) could not be tested (hemolyzed, lipemic, or icteric serum), and were therefore excluded. The remaining 1194 acute serum samples were tested for CHIKV by RT-PCR and ELISA (IgM/IgG). A total of 923/1194 (77.3%) convalescent blood samples were collected during follow-up visits and laboratory tested for CHIKV IgG antibodies. The median number of days between acute presentation and obtaining a follow-up convalescent sample was 17 (IQR = 15–22). Of the 923 convalescent blood samples collected, 30 samples (3.3%) were excluded due to quality issues (hemolyzed, lipemic, or icteric serum) impacting laboratory testing, and 158 samples (17.1%) were unable to undergo laboratory testing due to supply shortages. Figure 1 provides a flow-chart illustration of participant enrollment, acute and convalescent visits, and associated blood samples collected and tested.

Among the 1194 AUFI samples tested, a total of 331 samples were CHIKV positive (27.7%). Of the 331 positive cases detected, 81 (24.5%) were confirmed through RT-PCR, 129 (39.0%) by IgM ELISA, and 121 (36.6%) by IgG acute and convalescent ELISA. Of the 1194 AUFI samples, 1017 (85.2%) samples were additionally tested for dengue using RT-PCR, with six positive CHIKV samples detected with DENV coinfection (four cases DENV-2 and two cases DENV-4). Of these coinfections, four CHIKV infections were detected by RT-PCR, and two by IgM ELISA. 177 samples were not tested for dengue virus due to limitations in testing supplies and sufficient serum quantity of some participants during the testing period. All coinfections were detected in Kandal province, located adjacent to the Cambodian capital Phnom Penh.

Table 1 provides a demographic and clinical summary of study subjects evaluated for CHIKV during the 2020 outbreak. Highest CHIKV positivity testing proportions were recorded in May and November (40.2%, 39.4% respectively; p < 0.001). Positivity proportion among adults (age ≥ 18 years old) were significantly higher than children [32.9%, 20.1% respectively; p < 0.001, OR (95% CI) 1.9 (1.4–2.6)]. There were no statistical significance associations between gender, education, or occupation and testing positive for CHIKV. Further data summarizing CHIKV laboratory testing results and analysis of symptoms observed as proportions of CHIKV positive and CHIKV negative study subjects are provided as an Additional file 1.

**Table 1:** Characteristics of subjects tested for CHIKV during the outbreak in 2020

Parameter	Total tested	Positive		Negative		Sig	OR (95% CI)
		N	%	N	%		
Number of subjects	1194	331	27.7	863	72.3		
Month							
January	147	19	12.9	128	87.1	< 0.001	
February	140	40	28.6	100	71.4		
March	134	36	26.9	98	73.1		
April	94	25	26.6	69	73.4		
May	97	39	40.2	58	59.8		
June	51	4	7.8	47	92.2		
July	125	32	25.6	93	74.4		
August	102	35	34.3	67	65.7		
September	77	21	27.3	56	72.7		
October	91	29	31.9	62	68.1		
November	71	28	39.4	43	60.6		
December	65	23	35.4	42	64.6		
Season							
Rainy season (May–Oct)	543	160	29.5	383	70.5	0.220	1.2 (0.9–1.5)
Dry season (Nov–Apr)	651	171	26.3	480	73.7		
Site							
Battambang	88	7	8.0	81	92.0	< 0.001	
Kampong Speu	303	60	19.8	243	80.2		
Kandal	507	166	32.7	341	67.3		
Kratie	193	82	42.5	111	57.5		
Preah Vihear	61	8	13.1	53	86.9		
Sihanoukville	42	8	19.0	34	81.0		
Area							
Urban	810	226	27.9	584	72.1	0.844	1.0 (0.8–1.4)
Rural	384	105	27.3	279	72.7		
Age							
Mean (SD)	25.7 (± 17.8)	28.3 (± 17.3)		24.7 (± 17.9)		0.002	
Median	22.5	25.0		21.0			
IQR	11–38	14–40		10–36			
Age group							
Adult (age ≥ 18 years)	712	234	32.9	478	67.1	< 0.001	1.9 (1.5–2.6)
Children	482	97	20.1	385	79.9		
02–05 years	118	20	16.9	98	83.1	< 0.001	
06–17 years	364	77	21.2	287	78.8		
18–45 years	510	176	34.5	334	65.5		
46–65 years	174	48	27.6	126	72.4		
> 65 years	28	10	35.7	18	64.3		
Gender							
Male	589	168	28.5	421	71.5	0.543	1.1 (0.8–1.4)
Female	605	163	26.9	442	73.1		
Education							
No education	648	183	28.2	465	71.8	0.413	
Primary	238	58	24.4	180	75.6		
Secondary and higher	308	90	29.2	218	70.8		
Occupation							
Employed	182	51	28.0	131	72.0	0.915	1.0 (0.7–1.4)
Unemployed	1012	280	27.7	732	72.3		
Symptom							
Fever (100%)	1194	331	27.7	863	72.3	–	–
Headache	1153	323	28.0	830	72.0	0.235	1.6 (0.8–3.7)
Sore throat	927	244	26.3	683	73.7	0.05	0.7 (0.5–1.0)
Chills	885	259	29.3	626	70.7	0.042	1.4 (1.1–1.8)
Cough	877	219	25.0	658	75.0	< 0.001	0.6 (0.5–0.8)
Malaise	815	223	27.4	592	72.6	0.682	0.9 (0.7–1.2)
Muscle aches	493	151	30.6	342	69.4	0.061	1.3 (1.0–1.7)
Joint pain	332	109	32.8	223	67.2	0.015	1.4 (1.1–1.9)
Nausea	255	106	41.6	149	58.4	< 0.001	2.3 (1.7–3.0)
Vomit	132	57	43.2	75	56.8	< 0.001	2.2 (1.5–3.2)
Abdominal cramp	67	18	26.9	49	73.1	0.887	1.0 (0.5–1.7)
Shortness of breath	65	17	26.2	48	73.8	0.787	0.9 (0.5–1.6)
Lesion	62	36	58.1	26	41.9	< 0.001	3.9 (2.3–6.7)
Rash	32	10	31.3	22	68.8	0.643	1.2 (0.6–2.5)
Diarrhea	31	9	29.0	22	71.0	0.851	1.1 (0.5–2.3)
Seizure	10	1	10.0	9	90.0	0.225	0.3 (0.1–1.8)
Bloody urine	1	1	100.0	0	0.0	*–*	*–*
Bleeding	1	0	0.0	1	100.0	*–*	*–*
Jaundice	1	1	100.0	0	0.0	*–*	*–*

Most CHIKV positive individuals (280/331, 84.6%) reported having a fever 3 to 4 days prior to visiting a health facility for assessment. Symptoms including chills (259/885, 29.3%; p = 0.042; OR (95% CI) 1.4 (1.1–1.8)), joint pain (109/332, 32.8%; p = 0.015; OR (95% CI) 1.4 (1.1–1.9)), nausea (106/255, 41.6%; p < 0.001; OR (95% CI) 2.3 (1.7–3.0)), and vomiting (57/132, 43.2%; p < 0.001; OR (95% CI) 2.2 (1.5–3.2)) were all significant in CHIKV positive individuals. Lesions, which were defined in the study as the detection of bump, ulcer, abscess, or tumor on the patient’s skin, were also a significant symptom detected among CHIKV positive individuals (36/62, 58.1%; p < 0.001; OR (95% CI) 3.9 (2.3–6.7)). In addition to fever, more than 70% of CHIKV positive individuals also recorded additional symptoms including headache and sore throat; however, these were not statistically significant between CHIKV positive and negative individuals. A significant negative association between CHIKV positive individuals and cough was also recorded in the study (219/877, 25.0%; p < 0.001; OR (95% CI) 0.6 (0.5–0.8)). Of the 331 CHIKV positive individuals, 297 (89.7%) completed a follow-up assessment within 14 to 20 days of the acute visit. Of those, 27 (9.1%) reported ongoing joint pain-symptoms. No other symptoms were reported among positive CHIKV individuals during the follow-up assessments. Sixty-eight (68, 20.5%) CHIKV positive subjects were admitted to hospital during 2020. Forty-one (41, 12.4%) CHIKV positive subjects reported recent domestic travel defined as being outside of their respective home townships prior to acute assessment. No individuals in the study reported any recent international travel.

**Fig. 2:**
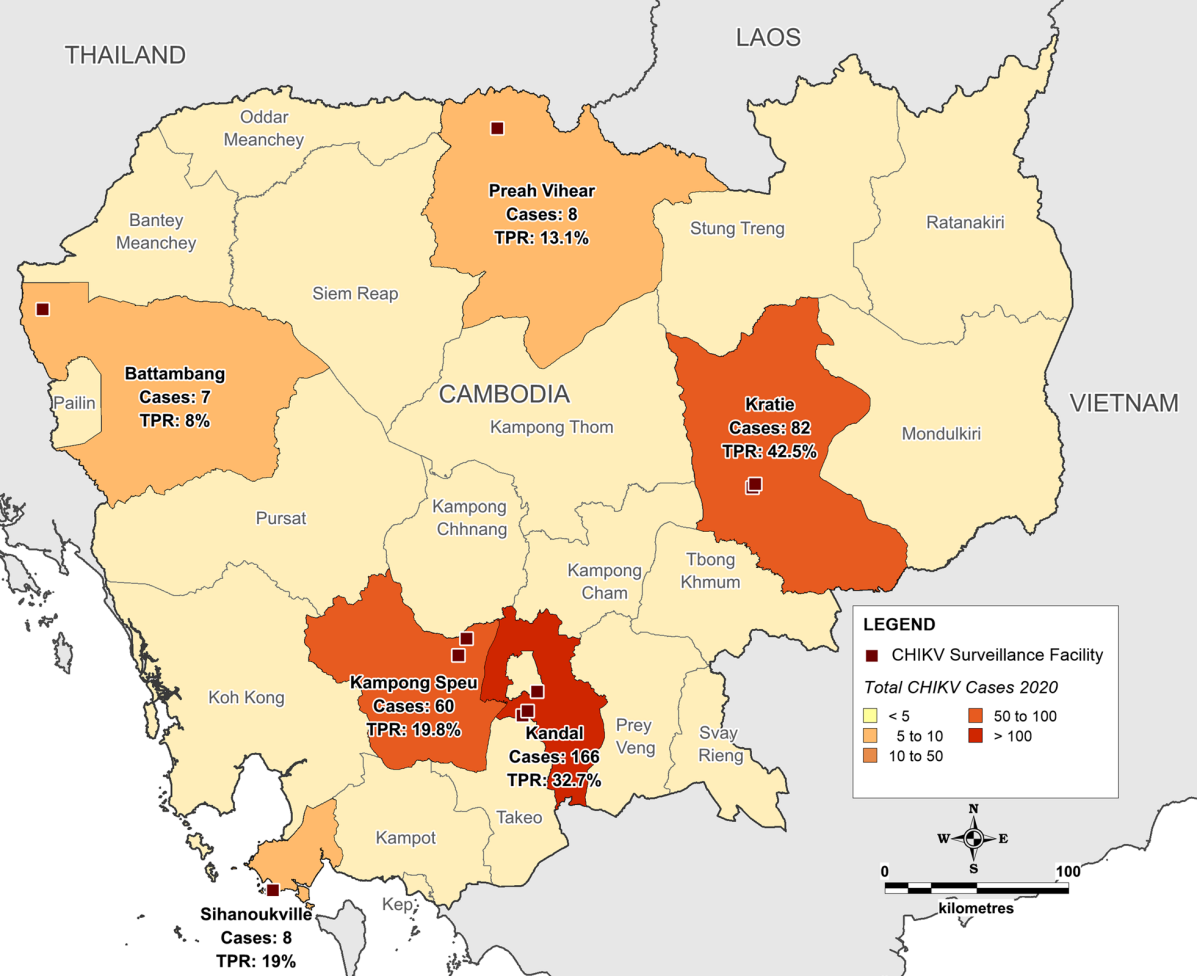
Location map illustrating surveillance facilities, and spatial distribution of positive chikungunya virus cases and test positivity rate (TPR) recorded by study site province in 2020. Custom map produced using MapInfo Professional v15.0.2 (Pitney Bowes Software Inc. 2015, Stamford, CT; https://www.pitneybowes.com)

Subjects residing in Kratie and Kandal provinces recorded the highest CHIKV test positivity proportions of all study site provinces (42.5% and 32.7% respectively; p < 0.001). The province where the greatest CHIKV test positivity proportion occurred were in the urban area of Kandal province (166, 50.2%) in southern Cambodia surrounding Phnom Penh, followed by the rural province of Kratie (82, 24.8%) in the northeast of the country, and the urban province of Kampong Speu (60, 18.1%), adjacent to Kandal. No significant difference in CHIKV test positivity proportion was identified between participants living in urban or rural areas, nor regarding seasonality between the dry and wet season. Figure 2 illustrates the spatial distribution of positive CHIKV cases recorded by study site province in 2020.

## Discussion

This study presents findings from a targeted health facility-based surveillance campaign to diagnose and characterize CHIKV positive patients during the 2020 Cambodian outbreak. Following a specific request from the Royal Cambodian Ministry of Health in 2020, CHIKV testing at pre-existing study sites was scaled-up, utilizing the established AUFI study resources and infrastructure as a platform to gather surveillance data to describe the disease epidemiology and characterize the clinical presentation and associated symptoms of CHIKV patients during the nationwide outbreak. Existing research highlights the need for increased granularity of surveillance data to better understand the disease dynamics of CHIKV [27]. Clinical and associated demographic data captured in this patient-level surveillance study provide insight not only into the 2020 Cambodia outbreak, but also serve to support strengthening the characterization of a broader epidemiological profile of CHIKV.

Consistent with previous findings, fever, joint pains (arthralgia), nausea, and vomiting were all found to be significantly associated with a positive case in this study [3, 28–31]. Severe arthralgia has been long associated with CHIKV, with the name “chikungunya” meaning to “walk bent over” in Makonde language in Tanzania, in reference to this symptom [28, 32]. Of note, however, while rash was not significantly associated with CHIKV in this study, there was a strong association recorded of CHIKV positive patients presenting with lesions. While not as commonly associated with CHIKV as fever and arthralgia, previous studies have also reported comparatively high rates of skin lesions, particularly among symptomatic adults [33]. These significant and commonly reported symptoms, combined with the additional finding in this study of a negative association of cough among AUFI patients and CHIKV, supports the classification of clinical criteria for identifying potential positive cases when using a syndromic approach in lower resource settings to create a differential diagnosis. Such criteria may be particularly useful during the initial stages of an outbreak to aid health workers to identify potential suspected CHIKV cases and refer patients for further testing and laboratory confirmation.

Key demographic findings from this study include an age-specific association with CHIKV positive patients. While no other demographic associations such as gender, education, or employment status were recorded, adults over the age of 18 were more likely to be CHIKV positive in the study as opposed to children. These data are consistent with similar studies that associate adults and young infants with a higher tendency to exhibit symptoms from CHIKV compared to children [33, 34]. Given the design of this study, which targeted symptomatic AUFI patients presenting at health facilities, and excluded children under 2 years of age, this may provide reasoning for the age-specific association with CHIKV positive patients, although further investigation into additional potential factors, such as job exposure, may also be warranted.

At the provincial level, the highest total cases (and second highest test positivity proportion) recorded as part of this study in 2020 were in the most populated and urbanized study site province of Kandal, an area surrounding the capital of Phnom Penh [35]. Of note, however, was the rural province of Kratie in northeastern Cambodia, bordering Vietnam, recording the highest test positivity proportion in the study, highlighting the impact of the outbreak in this more rural region of the country during 2020, and suggesting high levels of CHIKV transmission within a lesser developed location as well. These findings, together with the detection of positive CHIKV cases across all study provinces, indicate the widespread geographic distribution of CHIKV during the 2020 Cambodia outbreak, and highlights the need to target relevant public health response measures in both urban and rural settings during outbreak events, wherever local transmission is possible.

During a similar timeframe across the greater SEA region, documented CHIKV outbreaks were also reported in Thailand and Myanmar throughout 2018–2019 [36–40]. Additionally, in early 2020 in response to the COVID-19 pandemic, the Government of Thailand announced a plan to close their border with Cambodia, resulting in thousands of Cambodian nationals working in Thailand to return to Cambodia through several border checkpoints in the northwestern province of Banteay Meanchey. Subsequent surveillance reports highlighted the impact of the Chikungunya outbreak in northwestern Cambodian bordering Thailand (outside of the AUFI surveillance study area) during 2020 [41]. Limited data are available, however, on CHIKV transmission in neighboring countries to the east in Vietnam and northeastern Laos during this period. While no subjects assessed in this study reported recent international travel, further investigation to associate any connection of the 2020 Cambodia CHIKV outbreak to other documented outbreaks in SEA would be beneficial; and similarly, given the CHIKV cases detected in central and eastern Cambodia as part of this study, any potential impact or transmission events occurring in the adjacent border regions of Vietnam during this time. The multinational impact of CHIKV in 2020 highlights the need for coordinated surveillance networks that can accurately detect and report outbreaks as quickly and efficiently as possible.

Results from this study highlight multiple challenges associated with the diagnosis of CHIKV, particularly in the context of diagnosis at the point of care [19, 42]. Of note in this study were a relatively large number of RT-PCR-negative samples that met the serologic definition of a positive CHIKV case. While it is possible that potential issues such as RNA degradation and primer/probe mismatches can impact RT-PCR results, given strict protocols adhered to in this study as part of sample processing and laboratory testing procedures, these are considered unlikely causal factors. Given RT-PCR requires adequate RNA copies to achieve detection, a false negative test could occur even with the presence of symptoms if a patient presents for clinical evaluation when the molecular assay is below the limit of detection. Additionally, the results need to be interpreted within the limit of detection as previously described for this assay [24]. As such, sampling too late (or too early) to detect enough RNA copies was likely to have impacted these results. These findings demonstrate clear challenges associated with relying solely on RT-PCR testing and the potential to miss recent infections, particularly in the context of passive case detection of individuals presenting at health care facilities. Surveillance that incorporates both RT-PCR and serological testing (IgM (acute) positives and IgG seroconversions), as conducted in this study, provides a comprehensive approach to understanding overall disease prevalence and evaluating outbreaks.

In the absence of effective and readily available rapid diagnostics to aid point-of-care and field-based testing for CHIKV, surveillance-based research into the clinical characterization of suspected CHIKV infection provides essential data to support the management of both individual acute illness events, as well as the broader control of disease transmission within a community. Detailed patient-level data captured in this study, including clinical characteristics and epidemiological associations, as well as information on coinfections such as DENV, serves to strengthen the evidence base and enhance diagnostic decision making to help guide the identification of suspected CHIKV cases, supporting health practitioners to consider infection and seek appropriate laboratory confirmation. Similarly, utilizing these data to support increasing the awareness and knowledge of Chikungunya, both among health care professionals and general community would likely be of benefit to support control efforts. Incorporating molecular analysis into the surveillance framework to support the identification of CHIKV genotypes circulating in Cambodia would additionally serve to strengthen the epidemiological understanding of transmission.

Given the potential long-term impacts of CHIKV on population health [7, 9–12], together with the somewhat limited availability of CHIKV-specific surveillance data within SEA [3, 19], the detailed patient level surveillance data captured in this study suggests the need for further longitudinal research to characterize the contemporary epidemiology and transmission dynamics. As the geographic distribution of CHIKV transmission continues to increase, along with the expected frequency of outbreak events [3, 6], strengthening the understanding and documenting the long-term impacts of this disease within Cambodia and SEA region is essential.

As this study utilized passive surveillance data that focused on symptomatic AUFI patients presenting at health facilities, a limitation of this study is a lack of detection of asymptomatic and mildly symptomatic cases during the outbreak. Given relatively small samples collected at some locations, including Battambang, Preah Vihear, and Sihanoukville, there was limited power to conduct sub-analyses such as spatial analysis across site locations in this study. Additionally, limited testing for CHIKV was conducted at the study sites prior to 2020 and was limited to the targeted IgM and/or IgG assay testing of suspected cases, impacting the capacity for comparative analysis regarding test positivity proportion and raw case numbers from 2020 and previous years. Documented complexities associated with serological testing and potential cross-reactivity with DENV where co-circulation occurs [43] should also be considered in the context of this study. It should also be noted that no phylogenetic analysis was conducted in this study.

## Conclusion

This study characterizes the key clinical, demographic, and spatiotemporal characteristics of patients testing positive to CHIKV at study site health facilities during the 2020 Cambodia outbreak. Given the significant challenges associated with the availability of granular surveillance data to support the characterization of CHIKV, together with rapid diagnostic limitations, case data captured from this study has provided useful high-resolution and region-specific epidemiologic data and laboratory results on this re-emerging disease. As the geographic range and expected frequency of CHIKV outbreak continues to increase, strengthened disease-specific surveillance and an increased capacity to characterize, identify and diagnose suspected CHIKV infections will be essential to curb transmission.

## Supplementary Information

**Additional file 1.** Summary data of CHIKV laboratory testing results and analysis of symptoms observed as proportions of CHIKV positive and CHIKV negative study subjects. Further data summarizing CHIKV laboratory testing results and analysis of symptoms observed as proportions of CHIKV positive and CHIKV negative study subjects.

## Data Availability

The datasets generated during and/or analyzed during the current study are available from the corresponding author on reasonable request.

## Abbreviations

AUFI: Acute undifferentiated febrile illness
CHIKV: Chikungunya virus
CIs: Confidence intervals
DENV: Dengue virus
DALYs: Disability adjusted life years
ECSA-IOL: East-Central-South-African Indian Ocean Lineage
ELISAs: Enzyme-linked immunosorbent assays
IgG: Immunoglobulin G
IgM: Immunoglobulin M
NIPH: National Institute of Public Health
NAMRU-2: Naval Medical Research Unit-2
RT-PCR: Reverse transcription-polymerase chain reaction
RNA: Ribonucleic acid
US: United States
