# Supplementary material for: Clinical and epidemiologic evaluation of a 2020 chikungunya outbreak in Cambodia
Source: BMC Infect Dis. 2022 Dec 17;22:949. doi: 10.1186/s12879-022-07936-9 (PMC9758031; doi:10.1186/s12879-022-07936-9)
Supplement: Supplementary file 1 — Additional file 1. Summary data of CHIKV laboratory testing results and analysis of symptoms observed as proportions of CHIKV positive and CHIKV negative study subjects. Further data summarizing CHIKV laboratory testing results and analysis of symptoms observed as proportions of CHIKV positive and CHIKV negative study subjects. [file 12879_2022_7936_MOESM1_ESM.pdf]

Additional File 1: Summary data of CHIKV laboratory testing results and analysis of symptoms observed as proportions of CHIKV positive and CHIKV negative study subjects

Summary of CHIKV laboratory testing results

**CHIKV testing**	**Samples collected**	**Tested**	**Positive n (%)**
Chikungunya RT-PCR (acute)	1194	1194	81 (6.8)
Chikungunya ELISA*			
IgM (acute)	1194	713	138 (19.4)
IgG (acute-convalescent)	923	735	181 (24.6)

** Randomized samples tested due to supply shortages*

Summary of symptoms observed as proportions of CHIKV positive and CHIKV negative study subjects

Parameter	CHIKV positive	Proportion of CHIKV Positive	CHIKV negative	Proportion of CHIKV Negative	Sig	OR (95%CI)
**Number of subjects**	**331**	**100%**	**863**	**100%**		
**Symptoms reported**						
Fever (100%)	331	100%	863	100%	-	-
Headache	323	97.6%	830	96.2%	0.235	1.6 (0.8-3.7)
Sore throat	244	73.7%	683	79.1%	0.050	0.7 (0.5-1.0)
Chills	259	78.2%	626	72.5%	**0.042**	**1.4 (1.1-1.8)**
Cough	219	66.2%	658	76.2%	<0.001	0.6 (0.5-0.8)
Malaise	223	67.4%	592	68.6%	0.682	0.9 (0.7-1.2)
Muscle aches	151	45.6%	342	39.6%	0.061	1.3 (1.0-1.7)
Joint pain	109	32.9%	223	25.8%	**0.015**	**1.4 (1.1-1.9)**
Nausea	106	23.0%	149	17.3%	**<0.001**	**2.3 (1.7-3.0)**
Vomit	57	17.2%	75	8.7%	**<0.001**	**2.2 (1.5-3.2)**
Abdominal cramp	18	5.4%	49	5.7%	0.887	1.0 (0.5-1.7)
Shortness of breath	17	5.1%	48	5.6%	0.787	0.9 (0.5-1.6)
Lesion	36	10.9%	26	3.0%	**<0.001**	**3.9 (2.3-6.7)**
Rash	10	3.0%	22	2.5%	0.643	1.2 (0.6-2.5)
Diarrhea	9	2.7%	22	2.5%	0.851	1.1 (0.5-2.3)
Seizure	1	0.3%	9	1.0%	0.225	0.3 (0.1-1.8)
Bloody urine	1	0.3%	0	0.0%	-	-
Bleeding	0	0.0%	1	0.1%	-	-
Jaundice	1	0.3%	0	0.0%	-	-
